# Prevalence of tuberculosis, HIV, and TB-HIV co-infection among pulmonary tuberculosis suspects in a predominantly pastoralist area, northeast Ethiopia

**DOI:** 10.3402/gha.v8.27949

**Published:** 2015-12-18

**Authors:** Mulugeta Belay, Gunnar Bjune, Fekadu Abebe

**Affiliations:** 1 Aklilu Lemma Institute of Pathobiology, Addis Ababa University, Addis Ababa, Ethiopia; 2 Department of Community Medicine, Institute of Health and Society, Faculty of Medicine, University of Oslo, Oslo, Norway

**Keywords:** tuberculosis, HIV, co-infection, pastoralists, Afar, Ethiopia

## Abstract

**Background:**

TB-HIV co-infection is one of the biggest public health challenges in sub-Saharan Africa. Although there is a wealth of information on TB-HIV co-infection among settled populations in Africa and elsewhere, to our knowledge, there are no published reports on TB-HIV co-infection from pastoral communities. In this study, we report the prevalence of TB, HIV and TB-HIV co-infection among pulmonary TB suspects in the Afar Regional State of Ethiopia.

**Design:**

In a cross-sectional study design, 325 pulmonary TB suspects were included from five health facilities. Three sputum samples (spot-morning-spot) were collected from each participant. Sputum samples were examined for the presence of acid fast bacilli using Ziehl–Neelsen staining method, and culture was done on the remaining sputum samples. Participants were interviewed and HIV tested.

**Results:**

Of the 325 pulmonary TB suspects, 44 (13.5%) were smear positive, and 105 (32.3%) were culture positive. Among smear-positive patients, five were culture negative and, therefore, a total of 110 (33.8%) suspects were bacteriologically confirmed pulmonary TB patients. Out of 287 pulmonary TB suspects who were tested for HIV infection, 82 (28.6%) were HIV positive. A significantly higher proportion of bacteriologically confirmed pulmonary TB patients [40 (40.4%)] were HIV co-infected compared with patients without bacteriological evidence for pulmonary TB [42 (22.3%)]. However, among ethnic Afar pastoralists, HIV infections in smear- and/or culture-negative pulmonary TB suspects [7 (7.6%)] and bacteriologically confirmed pulmonary TB patients [4 (11.8%)] were comparable. On multivariable logistic regression analysis, Afar ethnicity was independently associated with low HIV infection [OR=0.16 (95% CI: 0.07–0.37)], whereas literacy was independently associated with higher HIV infection [OR=2.21 (95% CI: 1.05–4.64)].

**Conclusions:**

Although the overall prevalence of TB-HIV co-infection in the current study is high, ethnic Afars had significantly lower HIV infection both in suspects as well as TB patients. The data suggest that the prevalence of HIV infection among Afar pastoralists is probably low. However, population-based prevalence studies are needed to substantiate our findings.

Despite the implementation of a widely adopted strategy to control tuberculosis (TB), the disease remains a major public health problem, particularly in developing countries (1). In 2013, an estimated 9 million individuals developed TB and 1.5 million died from the disease (2). Similarly, the HIV epidemic remains a huge global challenge, and in 2012, an estimated 35.3 million people were living with the virus, whereas 1.6 million died (3). The prevalence of TB-HIV co-infection widely varies; the prevalence of HIV among TB patients ranged from 3.8 to 72.3%, whereas the prevalence of TB among HIV-positive patients ranged from 2.9 to 64.5% (4). The African region accounted for 75% of the estimated number of HIV-positive incident TB cases (2). Although many countries have made considerable progress in addressing the TB-HIV co-morbidity, global targets for HIV testing among TB patients and provision of antiretroviral therapy to those who are HIV positive have not been reached (2). HIV markedly increases the risk of progression to active TB (5) and the mortality associated with TB. Likewise, TB is a leading killer among HIV-positive individuals (4); TB also hastens the progression of HIV disease, increasing the risk of developing other opportunistic infections (6). Thus, the influence of each infection on the other's natural history and pathogenesis has enhanced the magnitude of TB/HIV epidemic (7). The number of people dying from HIV-associated TB has been falling since 2003; however, there were still 360,000 deaths from HIV-associated TB across the globe in 2013 (2).

Although several studies reported TB-HIV co-infection in many African countries including Ethiopia (8–10), to our knowledge, there are no reports on TB-HIV co-infection from pastoralist areas. Pastoralists constitute a substantial proportion of the population of Africa. Ethiopia, having the largest cattle population in Africa (11), is also home to an estimated 10 million pastoralists, about 12% of the total population (12). Pastoralists live in drought prone, arid areas and often face serious food shortages (13) and stress which are important risk factors for TB. Besides, their mobility could be a driving factor for HIV transmission as well as a challenge for intervention efforts. Here, we report for the first time, the prevalence of TB-HIV co-infection among pulmonary TB suspects as well as TB patients in the Afar Region, a predominantly pastoral region in the northeast Ethiopia.

## Materials and methods

### Study area

The study area has been described elsewhere (14). Briefly, the study was conducted in two public (Awash Health Center and Dubti Hospital) health facilities in the Afar Region and three private (Selam hospital, Bati Hospital, and Amir Higher Clinic) health facilities in Dessie Town. The reason for including these private health facilities is because they provide diagnostic services for a substantial number of TB patients coming from the Afar Region. Laboratory work was performed at Aklilu Lemma Institute of Pathobiology TB laboratory, Addis Ababa University.

### Study design and study participants

A health institution based cross-sectional study was conducted in the five health facilities from September 2009 to March 2010. Pulmonary TB suspects (≥18 years of age) who were residents of the Afar Region during the study period and had a cough lasting for 2 weeks or more were included consecutively. A sample size of 339 was estimated using a prevalence of 33% for bacteriologically confirmed pulmonary TB (15), margin of error of 5% and 95% CI.

### Data collection and sputum culture

Using a structured, pre-tested questionnaire, patients were interviewed by trained laboratory technicians on basic sociodemographic characteristics and their symptoms. Then, as previously described (16), Ziehl–Neelsen staining was done on three sputum samples (spot-morning-spot) collected from each participant. The remaining sputum samples were stored at 4°C and subsequently transported to Aklilu Lemma Institute of Pathobiology TB laboratory within 1 week. Subsequently, the three sputum samples from each participant were pooled, processed, and cultured according to WHO guidelines (17). Briefly, sputum samples were homogenized and decontaminated with equal volume of 4% NaOH and centrifuged at 3,000 rpm for 15 min. The supernatant was poured off while the sediment was neutralized with 0.1N HCL using phenol red as an indicator. Two to four loopfuls of the sediment were inoculated in four slopes of Lowenstein–Jensen medium, and inspection of media was done every week to monitor the growth until 8 weeks. Ziehl–Neelsen stain was done to detect acid fast bacilli.

### HIV test

HIV testing was done using KHB (Shanghi Kehua Bio-engineering, Ltd, Shanghai, China) as a screening test, HIV1/2 STAT-PAK^®^ ASSAY (CHEMBIO Diagnostic systems, Inc., Medford, NY, USA) as a confirmatory test, and Uni-Gold TM (Trinity Biotech, Jamestown, NY, USA) as a tie-breaker according to the national algorithm (18). HIV testing was done after counseling, and patients with HIV infection were referred to HIV care and treatment clinics after post test counselling.

### Data analysis

Data were entered into EpiData version 3.1 and exported into SPSS for Windows version 20 for further analysis. Univariable analysis followed by multivariable analysis (logistic regression) was done to identify independent predictors of dependent variables. Variable selection was based on prior knowledge from literature and biological plausibility. The outcome variables were HIV infection and bacteriologically confirmed TB. A *p*-value for a two-tailed test less than 0.05 was considered as statistically significant.

### Ethical consideration

The study has been ethically approved by the National Research Ethics Review Committee from Ethiopia and the Regional Committees for Medical Research Ethics – South East Norway (Regionale Komiteer for medisinsk og helsefaglig forskningsettik, Sør-Øst) from Norway before commencing the study. Written informed consent was obtained from all study participants before the interview and sample collection.

## Results

### Sociodemographic characteristics

A total of 339 pulmonary TB suspects were included in this study. However, 14 participants were excluded because of poor quality of sputum, and hence 325 (95.9%) were included for analysis. The sociodemographic characteristics of study participants have been summarized in Table 1. About 75% of the study participants were below 45 years of age with a median age of 30 years (interquartile range: 25–45). Males constituted 58.8% of the study participants. Pastoralists represented 150 (46.2%) of the study participants.

**Table 1:** Sociodemographic characteristics of pulmonary TB suspects in northeast Ethiopia

Characteristics	Count (%) (*N*=325)
Age	
18–24	76 (23.4)
25–44	166 (51.1)
> 44	83 (25.5)
Sex	
Male	191 (58.8)
Female	134 (41.2)
Marital status	
Single	106 (32.6)
Married	195 (60.0)
Widowed	10 (3.1)
Divorced	14 (4.3)
Residence	
Urban	183 (56.3)
Rural	142 (43.7)
Religion	
Muslim	239 (73.5)
Christian	86 (26.5)
Ethnicity	
Afar	146 (44.9)
Amhara	128 (39.4)
Others	51 (15.7)
Literacy	
Cannot read and write	184 (56.6)
Can read and write	141 (43.4)
Occupation	
Pastoralist	150 (46.2)
Non-pastoralist	175 (53.8)

### Prevalence of smear-/culture-positive pulmonary tuberculosis and associated factors

Among the 325 pulmonary TB suspects, a total of 105 (32.3%) were identified as pulmonary TB patients based on culture results. Among the 105 culture positives, 39 (37.1%) were diagnosed as smear-positive pulmonary TB patients at health facilities. Five more suspects who were reported as smear positive turned out to be culture negative. Therefore, the prevalence of smear-positive pulmonary TB patients among 325 pulmonary TB suspects was 13.5%. Based on bacteriology results (direct smear and culture), a total of 110 (33.8%) were diagnosed as pulmonary TB patients.

Results of analysis of the association between sociodemographic and other selected variables with pulmonary TB are summarized in Table 2. On logistic regression analysis, male sex, age group 31–45 years, and HIV infection were independently associated with pulmonary TB. The odds of having pulmonary TB among female suspects was 52% [OR=0.48 (95% CI: 0.27–0.86)] less compared with males, and the age group 31–45 years had a 56% reduction in pulmonary TB compared with those less than 31 years of age. On the contrary, the odds of having pulmonary TB among HIV-positive pulmonary TB suspects was significantly higher [2.65 (95% CI: 1.41–4.97)] compared with the corresponding odds among HIV-negative pulmonary TB suspects.

**Table 2:** Association of pulmonary TB and sociodemographic characteristics, northeast Ethiopia

Characteristics	Pulmonary TB, number (%)	Crude OR (95% CI)	Adjusted OR (95% CI)
Age (in years)			
18–30	73 (40.6)	1	1
31–45	19 (24.4)	0.47 (0.26–0.86)	0.44 (0.22–0.87)
> 45	18 (26.9)	0.54 (0.29–1.00)	0.70 (0.34–1.47)
Sex			
Male	74 (38.7)	1	1
Female	36 (26.9)	0.58 (0.36–0.94)	0.48 (0.27–0.86)
Residence			
Rural	44 (31.0)	1	1
Urban	66 (36.1)	1.26 (0.79–2.00)	1.54 (0.73–3.25)
Literacy			
Cannot read and write	58 (41.1)	1	1
Can read and write	52 (28.3)	1.77 (1.12–2.82)	1.10 (0.55–2.23)
Occupation			
Pastoralist	45 (30.0)	1	1
Non-pastoralist	65 (37.1)	1.38 (0.87–2.19)	0.69 (0.32–1.49)
Ethnicity			
Afar	40 (27.4)	1	1
Amhara	50 (39.1)	1.70 (1.02–2.82)	1.48 (0.71–3.10)
Others	20 (39.2)	1.71 (0.86–3.34)	1.68 (0.70–4.02)
Raw milk ingestion			
No	62 (39.7)	1	1
Yes	48 (28.4)	0.62 (0.39–0.99)	0.71 (0.35–1.42)
Contact history^a^			
No	67 (32.2)	1	1
Yes	43 (36.8)	1.22 (0.76–1.97)	1.18 (0.68–2.03)
HIV infection			
Negative	59 (28.8)	1	1
Positive	40 (40.4)	2.4 (1.4–4.0)	2.65 (1.41–4.97)
BCG scar			
No	88 (33.6)	1	1
Yes	22 (34.9)	1.06 (0.60–1.89)	1.07 (0.54–2.12)

a Contact with known pulmonary TB patient.

### Prevalence of HIV infection and associated factors among study participants

A total of 287 (88.3%) participants agreed to be tested for HIV infection, and 82 (28.6%) were positive. The prevalence was comparable in males (26.4%) and females (31.5%) (Table 3). However, the peak HIV prevalence in females was at a younger age (18–24 years) compared with males (35–44 years) (data not shown). A very low proportion (8.7%) of HIV infection was found among ethnic Afars compared with the proportion of infection among other ethnic groups (Table 3).

**Table 3:** Association of HIV and sociodemographic characteristics, northeast Ethiopia

Characteristics	HIV positive number (%)	Crude OR (95% CI)	Adjusted OR (95% CI)
Age (in years)			
18–30	48 (30.6)	1	1
31–45	29 (42.0)	1.65 (0.92–2.96)	1.96 (1.00–3.87)
> 45	5 (8.2)	0.20 (0.08–0.54)	0.44 (0.15–1.31)
Sex			
Male	43 (26.4)	1	1
Female	39 (31.5)	1.28 (0.77–2.14)	1.81 (0.96–3.43)
Residence			
Rural	18 (15.0)	1	1
Urban	64 (38.3)	3.52 (1.95–6.35)	0.99 (0.43–2.28)
Literacy			
Cannot read and write	28 (17.2)	1	1
Can read and write	54 (43.5)	3.72 (2.17–6.38)	2.21 (1.05–4.64)
Ethnicity			
Amhara	54 (47.4)	1	1
Afar	11 (8.7)	0.11 (0.05–0.22)	0.16 (0.07–0.37)
Others	17 (36.2)	0.63 (0.31–1.27)	0.58 (0.28–1.22)
Religion			
Muslim	50 (23.9)	1	1
Christian	32 (41.0)	2.21 (1.27–3.84)	0.70 (0.35–1.38)
Occupation			
Pastoralist	15 (11.9)	1	1
Non-pastoralist	67 (41.6)	5.27 (2.83–9.84)	1.73 (0.75–4.03)

The association of HIV infection with sociodemographic variables was assessed using logistic regression analysis, and results are summarized in Table 3. On multivariable logistic regression, the proportion of HIV infection in TB suspects was significantly lower among ethnic Afars [OR=0.16 (95% CI: 0.07–0.37)]; on the other hand, being literate was independently associated with a significantly higher proportion of HIV infection [OR=2.21 (95% CI: 1.05–4.64)].

### HIV–TB co-infection

Among 110 TB patients, 99 were tested for HIV infection, of which 40 (40.4%) were positive for HIV. The proportion of HIV-positive participants among TB patients was significantly higher compared with the proportion of HIV infection (22.3%) among smear- and/or culture-negative pulmonary TB suspects (*p*=0.001). TB-HIV co-infection rates significantly varied among ethnic groups, and ethnic Afars had the lowest proportion (11.8%) of HIV-positive TB patients. Compared with ethnic Amharas, being ethnic Afar [OR=0.09 (95% CI: 0.02–0.41)] was independently associated with low TB-HIV co-infection after adjusting for age, sex, literacy, occupation, residence, and religion. The proportion of smear positivity among HIV co-infected pulmonary TB patients was significantly lower (22.9%) compared with the corresponding proportion (45.8%) among HIV-negative pulmonary TB patients (*p*=0.03).

## Discussion

The HIV epidemic has influenced the epidemiology of TB significantly. HIV is a well-known risk factor for progression to active TB among those infected with *Mycobacterium tuberculosis* (7). Studies reported the prevalence of HIV infection among TB patients as well as TB suspects in settled populations as reviewed in Gao et al. (4). However, to our knowledge, this is the first report on TB-HIV co-infection and the prevalence of TB and HIV among pulmonary TB suspects from a predominantly pastoral community.

In this study, among pulmonary TB suspects, 13.5% were diagnosed as smear-positive pulmonary TB patients in agreement with a previous report from a rural hospital (19), but a higher prevalence of smear-positive pulmonary TB was observed in Addis Ababa (20). The prevalence of pulmonary TB among pulmonary TB suspects based on smear microscopy and culture was found to be 33.8% in agreement with a previous finding in a tertiary hospital in Addis Ababa (15). Such a high TB prevalence among suspects might be related to delay in the diagnosis of TB as previously reported in the study area (21). Gender disparities with regard to TB have been documented, although the reason for such differences is not well known. In agreement with a previous report from Ethiopia (15), a significantly higher proportion of male pulmonary TB suspects were diagnosed with TB compared with females suspected of pulmonary TB. As reviewed by Holmes et al. (22), gender disparity in TB notification has been reported mainly in high TB burden settings.

The proportion of HIV infection among pulmonary TB suspects was 28.6% which is in agreement with a previous report from Addis Ababa (20). Interestingly, ethnic Afar pulmonary TB suspects had the lowest proportion of HIV infection compared with other ethnic groups, and such low prevalence has been previously documented among pregnant Afar women in the study area (23). Generally, the prevalence of HIV infection among TB suspects is believed to reflect the HIV prevalence in the general population, and our data may suggest a low HIV infection among ethnic Afar population. The relatively low HIV prevalence in ethnic Afars might be related to their geographical and cultural inaccessibility to the rest of the country. However, changes in the life style of ethnic Afars with a tendency of settlement as well as urbanization could facilitate the spread of HIV infection among this population.

In this study, the proportion of HIV positivity among pulmonary TB patients (40.4%) was comparable with a report from Ethiopia (9). However, HIV co-infection among TB patients in our study was higher compared with the co-infection rate reported in urban (20.2%) (24) as well as rural (18% and 19%) Ethiopia (25, 26). The average TB-HIV co-infection for Africa (32%) (7) is also less compared with the co-infection rate in the current study.

Although low compared with non-Afars, the prevalence of TB-HIV co-infection among indigenous Afar population with traditional culture is still high and worrisome for many reasons. First, TB-HIV co-infection is usually associated with urban poor rather than inaccessible remote regions with traditional ways of life such as cattle herding. Second, pastoralists have less access to education, health services, communication, and transportation and, therefore, poor knowledge and practices regarding prevention of HIV infection (27). Third, pastoralists are migratory people who move with their animals from season to season in search of water and pasture, which implies that the current TB control strategy (directly observed treatment, short course at fixed health facilities), antiretroviral therapy, and counseling and testing for controlling HIV infection, which are primarily designed for settled population, are not easily available to them as documented in other mobile populations (28). When such health services are available, they are easily interrupted because of seasonal migration with their animals, suggesting the need for designing a suitable TB/HIV control strategy pertinent to pastoral way of life to contain the spread of HIV infection (29).

The World Health Organization recommends integrated TB and HIV activities on the interventions needed to prevent, diagnose, and treat TB in people living with HIV. These include establishing and strengthening coordination mechanisms for delivering integrated TB and HIV services (1). However, pastoral communities lack basic health services and depend on traditional healers rather than health services provided by health authorities, which is usually meager and ineffective. Therefore, policy makers need to consider how new interventions can be adopted by TB and HIV control programs, and implemented on a sustainable basis in the pastoral communities to limit the spread of HIV infection.

The low prevalence of HIV among ethnic Afar pulmonary TB suspects and pulmonary TB patients may not necessarily reflect HIV prevalence among ethnic Afars, and therefore, a community-based study is needed to determine the population prevalence. Besides, not all health facilities were included in this study, and, hence, our findings may not be generalizable to all ethnic Afar pulmonary TB suspects and pulmonary TB patients in the region.

## Conclusions

Although the overall prevalence of TB-HIV co-infection in the current study is high, ethnic Afars had significantly lower HIV infection both in suspects as well as TB patients. These data suggest that the prevalence of HIV among Afar pastoralists is probably low. However, population-based prevalence studies are needed in designing intervention strategies to limit the spread of HIV infection among Afar pastoralists.
